# The relationship between social networking sites usage and psychological distress among undergraduate students during COVID-19 lockdown

**DOI:** 10.1016/j.heliyon.2020.e05695

**Published:** 2020-12-09

**Authors:** Tariq N. Al-Dwaikat, Mohammed Aldalaykeh, Wafa'a Ta'an, Mohammad Rababa

**Affiliations:** Jordan University of Science and Technology Faculty of Nursing, P.O. Box 3030, Irbid, 22110, Jordan

**Keywords:** Psychology, Undergraduate students, Anxiety, Stress, Depression, Psychological distress, Social networking sites

## Abstract

During the outbreak of the COVID-19 pandemic, interpersonal interactions are restricted to social networks. Undergraduate students are isolated in their homes and dorms. Loneliness is closely related to psychological distress. Fear of contracting the disease will worsen psychological distress. The purpose of this study was to assess the severity of depression, anxiety, and stress symptoms among undergraduate students and their relationships with social networking sites usage during the COVID-19 lockdown. An online survey was used to recruit 456 participants for this cross-sectional descriptive study. Self-reported questionnaires were used to collect data on students' demographics, depression, anxiety, stress, and social networking usage. The results revealed that the majority of students had symptoms of depression (74.1%), anxiety (59.6%), and stress (61.2%). Female students had higher depression and anxiety symptoms than males. Senior-level students' psychological distress symptoms were significantly different from those of junior level. The largest percentage of students (91.9%) used social networking sites for entertainment. The academic usage of social networking sites was negatively (*p* < .05) correlated with depression and stress scores, while entertainment usage was positively correlated with anxiety. Age was not found to be significantly correlated with psychological distress. Academic and entertainment use of social networking sites were successfully associated with psychological distress symptoms after controlling for demographics. During this unprecedented time of undergraduate students’ course of study, they were experiencing higher than average distress symptoms. These symptoms could be mitigated by continuing the regular academic activities and delivering the most accurate up-to-date information on the COVID-19 through social networking sites.

## 1 Introduction

During this critical time in the history of human beings, many people are suffering from psychological distress. Undergraduate students who are among the most dynamic groups are suffering too. Although students can communicate through social networking sites (SNS) and applications, they are still prone to psychological problems. More information needs to be collected to get a full picture of the psychological wellbeing of undergraduate students during the Coronavirus disease 2019 (COVID-19) pandemic and how the use of SNS relates to either improving or aggravating psychological distress symptoms.

The COVID-19 transmits from a person to another via respiratory droplets (Centers for Disease Control and Prevention, 2020), which makes it one of the fastest-growing communicable diseases in the history of the human being. As of December 9^th^, 2020, the COVID-19 affected more than 68 million persons, and resulted in more than 1.5 million deaths worldwide (Johns Hopkins Coronavirus Resource Center, 2020). The high transmissibility of the COVID-19 globally and the doubt about the suspected cases led many governments in the world to impose lockdowns on all schools and universities, in addition to many businesses. These decisions left many undergraduate students alone and sometimes away from their homes. The loneliness feelings among this group of youth may aggravate the psychological distress symptoms of depression, anxiety, and stress as well as other health problems such as eating disorders (Pantic, 2014). Gender differences in psychological distress symptoms were evident among undergraduate students; where females appeared to suffer more distress symptoms than males (Ishaku et al., 2018). McIntyre et al. (2018) found that socially isolated undergraduate students suffered from psychological distress at higher rates than their counterparts who are engaged in peer groups.

Social networking and verbal social skills could mitigate the feelings of loneliness and thus improving the overall mental health especially in youth females (Ishaku et al., 2018; Milner et al., 2016; Moeller and Seehuus, 2019). New technological advances such as smartphone mobile applications were found to be effective in reducing loneliness and improving overall mental health (Lim et al., 2019). On the other hand, social networking through these technologies and websites were associated with psychological and sleep problems (van der Velden et al., 2019). Sleep deprivation would increase psychological distress symptoms and results in poor cognitive, memory, and overall performance (Medic et al., 2017). These latter would be very influential in predicting academic performance among undergraduate students. In the worst-case scenario, loneliness and social isolation will lead to increased rates of suicidal ideation and attempts (Calati et al., 2019).

Social networking sites have both positive and negative effects on mental health and psychological distress symptoms (Yin et al., 2019). Social networking sites have been used consistently as a media to teach, learn, and exchange information, in addition to socializing and having fun (Gupta and Bashir, 2018). Students reported that they are more connected to their families and friends using social networking sites, however, they neglected their study and wasted their time in socializing (Hamade, 2013). Social network sites were found to increase the disruptions and consequently affecting students' academic performance negatively (Karpinski et al., 2013). On the other hand, the use of these social networks was found to be improving interactive and collaborative learning (Al-rahmi and Othman, 2013). Social networks were beneficial in increasing discussions, chatting, file sharing which lead to improve the learning process and make it a more enjoyable and entertaining experience and thus increasing students’ knowledge (Eid and Al-Jabri, 2016).

The evaluation of social networking usage among undergraduate students should be approached cautiously. Social network usage is beneficial in socializing and keeping up with local and global events such as the current COVID-19 pandemic and thus improving psychological wellbeing. However, students’ performance at their schools and colleges could be affected negatively. Further research, using large samples, to examine the relationships between social networks usage and psychological distress symptoms was warranted (Morciano et al., 2016).

To date, this is the first study that assess the psychological status of undergraduate students during the COVID-19 pandemic and how it relates to their use of SNS. Despite the preliminary nature of the study, it contributes to a better understanding of factors influencing the psychological wellbeing of the students during times of emergency and disasters like the COVID-19 pandemic. Accordingly, the purpose of this study was to assess the severity of depression, anxiety, and stress symptoms among undergraduate students and their relationships with SNS usage during the COVID-19 lockdown. During this unprecedented time, it is not known yet how the lockdown affects undergraduate students' usage of social networks and how that usage relates to their psychological wellbeing. The relationships between these variables has not been clear enough to draw conclusions on the nature and direction of these relationships. In addition, the spread of the COVID-19 changes the formula and creates fear among undergraduate students about their health, academic performance, and generally, their future. Thus, it would be crucial to evaluate how SNS usage affects students’ psychological wellbeing during this difficult time.

## 2 Methods

### 2.1 Design and sample

A cross-sectional quantitative descriptive design was used to recruit 456 undergraduate students between April 3^rd^ and 9^th^, 2020. An online survey was used to collect the data. All undergraduate students in Jordan were eligible to participate in the study. Approval was obtained from the institutional review board of the principle's investigator's affiliated university. Participants were fully informed about the purpose of the study and the usage of their data. The principal investigator assured: maximizing the benefits and minimizing the risks and burdens on the participants, minimizing the physical, emotional, and financial harms, avoiding exploitation by assuring the participants that any information given will not be used against them. The students were assured that they have the right to accept or refuse participation or withdraw from the study without any negative consequence at any time, and the students were provided with a full description of the study. In addition, privacy and confidentiality were maintained using proper measures.

### 2.2 Measures

The data collection included three sections: demographic variables, the social network usage questionnaire, and the depression, anxiety, and stress scale.

#### 2.2.1 Demographics

The following demographics were assessed: age, sex, study level, and smoking to describe the participants’ characteristics and show similarities and variations among the study participants.

#### 2.2.2 Social Network Usage Questionnaire

Social Network Usage Questionnaire (SNUQ) was used to measure the social networking behaviors of undergraduate students (Gupta and Bashir, 2018). Content validity of the measure was supported, and exploratory factor analysis supported the construct validity. Gupta and Bashir conducted confirmatory factor analysis which revealed that the 19 items of SNUQ were factored into 4 subscales (*academic, socialization, entertainment, and informativeness*). The internal consistency reliability was very good; the Cronbach's α for the total scale was 0.83 (*N* = 420), in this study the Cronbach's α for the total scale was .88. The participants are asked to respond to SNUQ items using a 5-items Likert scale that ranges from (Never {0} to Always {5}). The total score is calculated by taking the average for all participants' responses and the average scores are used to calculate subscales scores too.

The SNUQ was translated from English into Arabic by a panel of three bilingual experts, then back translated into English by another professional translator. Then, the translated and original questionnaire were compared to assure that the translation did not affect the validity of the questionnaire.

#### 2.2.3 Depression, anxiety, and stress

Depression, anxiety, and stress were measured using the Depression, Anxiety, and Stress Scale-21 (DASS-21 (Lovibond and Lovibond, 1995), Lovibond and Lovibond, 1995). The DASS-21 is a 21-item brief scale designed to assesses the negative emotional state of depression, anxiety, and stress of individuals during the last week. The DASS-21 is composed of seven items for each subscale (anxiety, depression, and stress). The responses to these items range from 0 (*did not apply to me at all* – *never*) to 3 (*applied to me -very much, or most of the time – almost always*). The total score for each subscale is calculated by summing the scores for each item. The resulting totals are then classified into *normal, mild, moderate, severe,* or *extremely severe* (Lovibond and Lovibond, 1995). The validity of the DASS-21 was supported (Antony et al., 1998; Gloster et al., 2008). The internal consistency was supported by Cronbach's α for the depression (.94), anxiety (.87), stress (.91) (Antony et al., 1998). The DASS-21 was translated into Arabic and validated; the validation revealed that the Arabic version is psychometrically and theoretically supported (Moussa et al., 2001). In this study, the Cronbach's α′s for depression, anxiety, and stress were .88, .84, and .87 respectively.

### 2.3 Data analysis

All statistical analyses were conducted using the Statistical Package for the Social Sciences SPSS version 22 (IBM, Armonk, NY). For the descriptive and bivariate analysis, an α level of less than 0.05 was employed for the results to be significant. All continuous variables were tested for normality using the Kolmogorov–Smirnov test. The variables that violate the assumption of normality were transformed into more normally distributed scores. Participants' responses for each variable (measure) were evaluated; if the missing data per variables are 40% or more then participant's responses for that variable were deleted listwise (Raymond and Roberts, 1987). Missing data that are less than 40% were imputed by the participant's mean response of the present items for each specific measure (Raymond, 1986). Descriptive statistics were run to describe study participants' demographics and measures' total scores. Means, standard deviations, and ranges were used for continuous variables, while percentages and frequencies were used for categorical variables. Hierarchical multiple regression was used to build a model to assess for the correlates of psychological distress symptoms among undergraduate students controlling for demographics.

### 2.4 Outcome analyses

Bivariate analyses were conducted to determine associations between each of demographics, SNUQ scores, and DASS-21 scores. First, independent t-tests were employed to determine if there is an association between each of the followings: demographics, social network usage, and psychological distress (depression, anxiety, and stress). Second, the relationships between social network usage and all of the outcome variables (depression, anxiety, and stress) were examined. Pearson's product-moment correlation coefficients were used to identify the direction and strength of the relationship between SNUQ and DASS-21 scores.

### 2.5 Ethical approval and consent to participate

After obtaining the Jordan University of Science and Technology Institutional Review Board (IRB) approval (#223–2020) the participants were fully informed about the purpose of the study and the usage of their data. The principal investigator assured: maximizing the benefits and minimizing the risks and burdens to the participants, minimizing the physical, emotional, and financial harms, avoiding exploitation by assuring the participants that any information given will not be used against them. The students were assured that they have the right to accept or refuse participation or withdraw from the study without any negative consequence at any time, and the students were provided with a full description of the study. In addition, privacy and confidentiality were maintained using proper measures.

## 3 Results

**Figure 1:**
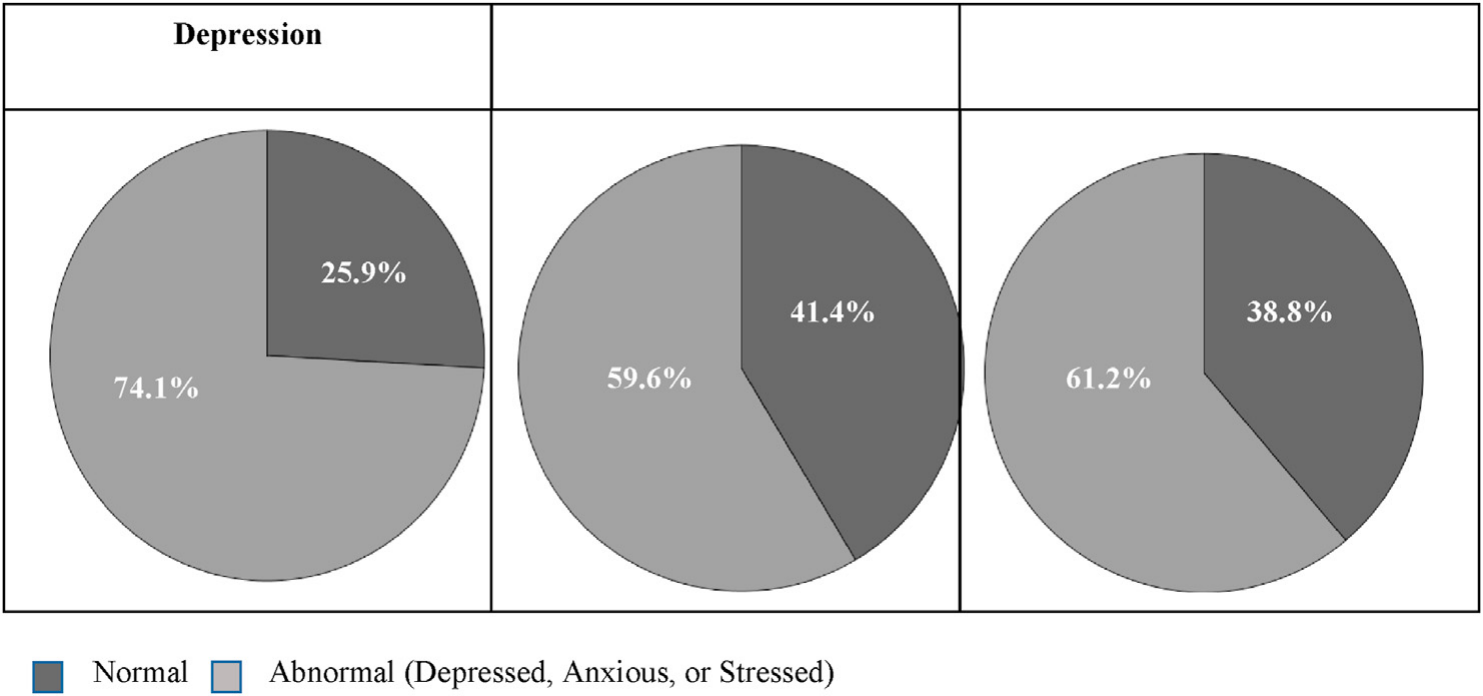
The frequency distribution of psychological distress symptoms for study participants.

A total of 456 undergraduate students participated in this study, no missing data were found. The normality assumption was checked; no violations were found among the variables. The Mean (M) age of the participants was 20.8 years (Standard Deviation [SD] = 2.24). The majority of participants were female (67.5%), in the upper division of their study (sophomores and seniors) (55.5%), and non-smokers (76.8%) (Table 1). The average levels of depression, anxiety, and stress were (9.1, SD= (5.7); 5.8, SD= (5.1); and 9.8, SD= (5.6)) respectively. According to Lovibond and Lovibond (1995), these average scores are interpreted as a moderate level of depression and mild levels of anxiety and stress. Figure 1 shows that the majority of the participants in this study were having symptoms of depression (74.1%), anxiety (59.6%), and stress (61.2%); these symptoms were ranging between mild and very severe form.

**Table 1:** Psychological distress symptoms differences between participants based on their demographics (n = 456).

Characteristic	N	%	Depression			Anxiety			Stress		
			Mean (SE)	*t*	*P*	Mean (SE)	*t*	*P*	Mean (SE)	*t*	*P*
**Sex**											
Male	148	32.5	8.7 (.48)	-.87	.39	5.0 (.40)	-2.24	.026	9.0 (.47)	-2.21	.028
Female	308	67.5	9.2 (.32)			6.1 (.29)			10.24 (.32)		
**Seniority in School**											
Lower Division	203	44.5	8.8 (.40)	-.86	.39	5.7 (.35)	-.23	.82	9.7 (.40)	-.27	.79
Upper Division	253	55.5	9.2 (.35)			5.8 (32)			9.9 (35)		
**Smoking**											
No	350	76.8	8.9 (.30)	-1.42	.16	5.6 (.26)	-1.14	.26	9.7 (.30)	-.97	.33
Yes	106	23.2	9.7 (.59)			6.3 (.53)			10.3		

N: number of participants, t: t-test value, p: significance level, SE: standard error.

T-tests (Table 1) revealed significant differences between female and male students in their scores of anxiety (*t* (454) = -2.24, *p* = .026) and stress (*t* (454) = -2.21, *p* = .028); where females reported higher anxiety and stress levels than males. However, there were no significant differences in depression scores between males and females. In addition, psychological distress symptoms did not differ significantly according to students’ seniority level or their smoking status.

Table 2 shows that the highest percentage of SNS usage purpose was for entertainment (91.9%), followed by informativeness (84.2%), academic (78.1%), and socialization (77.9%) among study participants. Table 3 shows the correlations between study variables. Pearson product correlations coefficients showed that age of the students was significantly negatively correlated with their usage of SNS for academic (*r* = -.10, *p* < .05) and entertainment purposes (*r* = -.10, *p* < .05), and positively with informativeness use of SNS (*r* = .14, *p* < .05). Depression scores of the students were significantly negatively correlated with academic (*r* = -.16, *p* < .01), informativeness (*r* = -.14, *p* < .01), and total usage of SNS (*r* = -.11, *p* < .05); which means that the academic, informative, and total uses of SNS played a role in partially relieving depression among study participants. In addition, entertainment use of the SNS was positively (*r* = .11, *p* < .05) correlated with anxiety scores, and academic use of SNS negatively correlated with (*r* = -.10, *p* < .05) with stress scores of the participants.

**Table 2:** The frequency distribution of social network sites usage for study participants (N = 456).

Frequency of Use	Type of Use	Academic	Socialization	Entertainment	Informativeness
Never		1.5%	2.4%	.4%	1.3%
Rarely		20.4%	19.7%	7.7%	14.5%
Sometimes		41.9%	46.7%	29.2%	40.6%
Often		28.3%	26.8%	42.1%	32.0%
Always		7.9%	4.4%	20.6%	11.6%

**Table 3:** Correlations between study variables.

	Age	Depression Scores	Anxiety Scores	Stress Scores	Socialization	Academic	Entertainment	Informativeness	Total SNUQ Score
Age	1	.03	.05	.03	-.03	-.10∗	-.10∗	.14∗∗	-.05
Depression Scores		1	.73∗∗	.85∗∗	-.08	-.16∗∗	.09	-.14∗∗	-.11∗
Anxiety Scores			1	.77∗∗	.01	-.08	.11∗	.01	-.003
Stress Scores				1	-.08	-.10∗	.08	-.06	-.07
Socialization					1	.48∗∗	.49∗∗	.58∗∗	.79∗∗
Academic						1	.44∗∗	.59∗∗	.86∗∗
Entertainment							1	.36∗∗	.70∗∗
Informativeness								1	.76∗∗
Total SNUQ Score									1

∗. Correlation is significant at *p* < 0.05, ∗∗. Correlation is significant at *p* < 0.01.

Table 4 shows that after controlling for demographics (age, sex, seniority, and smoking) academic and entertainment usages of SNS were successfully (*p* < .05) associated with depression, anxiety, and stress in this sample of students. The results of hierarchical multiple regression showed that 6% of the variance in depression was accounted for by the demographics and SNS usages (academic, socialization, entertainment, and informativeness); however, academic and entertainment usages of SNS were the only significant correlate of depression. Similarly, the study variables accounted for 3% of the anxiety, and academic and entertainment usages successfully associated with anxiety scores in this sample. In addition, study variables accounted for 3% of the stress, and academic and entertainment usages successfully associated with stress scores in this sample.

**Table 4:** Hierarchical multiple regression for correlates of psychological distress symptoms.

Model	Predictors	Depression		Anxiety		Stress	
		*β*	*t*	*β*	*t*	*β*	*t*
1	Age	.01	.11	.07	1.19	.03	.55
	Sex	.10	1.73	.17	3.17∗∗	.16	3.0∗∗
	Seniority Level	.04	.65	-.02	-.37	.001	.01
	Smoking	.10	1.96	.12	2.25∗	.11	2.11∗
2	Age	.05	.83	.07	1.19	.05	.93
	Sex	.09	1.67	.17	3.34∗∗	.15	2.88
	Seniority Level	-.004	-.08	-.04	-.65	-.03	-.60∗∗
	Smoking	.07	1.31	.10	1.88	.08	1.54
	Academic	-.19	-3.10∗∗	-.18	-3.0∗∗	-.14	-2.30∗
	Socialization	-.04	-.69	-.02	-.28	-.10	-1.54
	Entertainment	.23	4.08∗∗∗	.17	3.14∗∗	.19	3.37∗∗
	Informativeness	-.08	-1.30	.07	1.10	.01	.14
1	*F* (4, 451) = 1.395, *p* = .24, adjusted *R*^*2*^ = .003			*F* (4, 451) = 3.10, *p* = .016, adjusted *R*^*2*^ = .02		*F* (4, 451) = 2.55, *p* = .038, adjusted *R*^*2*^ = .01	
2	*F* (8, 447) = 4.56, *p* < .001, Δ*R*^2^ = .06			*F* (8, 447) = 3.60, *p* = .002, Δ*R*^2^ = .03		*F* (8, 447) = 3.32, *p* = .003, Δ*R*^2^ = .03	

∗*p* < .05, ∗∗*p* < .01, ∗∗∗*p* < .001.

## 4 Discussion

The findings of the current study revealed that the largest percentage of the surveyed students experienced psychological distress symptoms during this tough time. The reported average symptoms of depression, anxiety, and stress were ranging between mild and moderate. However, a significant percentage of the students suffered from a very severe form of depression (23.7%), anxiety (22.6%), and stress (15.4%). The experience of psychological distress symptoms among students in this study was much higher than Lovibond and Lovibond (1995) study that recruited 717 undergraduate students. These percentages are alarming and require professional consultation and immediate interventions. The psychological impact of the COVID-19 pandemic is being investigated and one of the factors that worsen this impact is the studentship status (Wang et al., 2020). Students are prone to psychological distress because they may be worried about contracting the disease, was afraid regarding their academic achievement and their future plans.

The results of this study also showed that female students had significantly higher levels of anxiety and stress symptoms, which may have resulted from their greater worries. This finding is consistent with a previous study which reported that female college student experienced higher levels of anxiety than their male counterparts; anxiety experiences were higher among females during their first and second year of study that was positively relates to body image and academic achievements (Gao et al., 2020). During the lockdowns of the colleges around the globe, academic performance could be best the correlate of anxiety, especially among female students. This latter statement needs further research and exploration.

Wang et al. (2020) found that trustworthy and up-to-date information were negatively correlated with psychological distress symptoms. In this study, the informativeness subscale of the SNS questionnaire scores was negatively correlated with the depression (*r* = -.14, *p* < .05). Students used SNS to navigate the information related to the COVID-19 pandemic spread across the globe through news and to get more information about the appropriate use of the personal protective equipment and other preventive measures. However, after controlling for sociodemographic variables, informativeness usage of SNS was not a successful correlate of depression. Thus, a cautionary interpretation of the negative association of the information with depression and other psychological symptoms should be taken into consideration. A study (Ho et al., 2020) recommended that a fight against COVID-19 fake news should be led by the governments by directing the citizens toward the appropriate sources of information such as the World Health Organization and the Centers for Disease Control and Prevention.

Although the largest percentage of the students in this study used SNS to entertain themselves by watching movies and sharing funny jokes or pictures, the entertainment use of the SNS was found to be significantly associated with psychological distress symptoms. However, the academic use of SNS such as attending academic discussion groups or consulting teachers was negatively associated with both depression and stress. These results indicated that students’ psychological distress could be lessened by keeping up with their regular academic activities. This relationship was bidirectional, which means that students who had lower psychological distress symptoms might have been able to utilize the SNS for academic purposes and lessen their use of the SNS for entertainment. Similar findings were shown by Hou et al. (2019) study; where they found that higher experience of psychological distress symptoms among undergraduates was associated with more problematic use of the SNS.

The apparently entertaining use of SNS had no association with psychological distress during this difficult time. Generally, the use of SNS for entertainment is suggested to have a negative impact on psychological distress symptoms (Liu et al., 2019). Thus, the use of SNS for academic purposes should be continued and future studies should focus on evaluating the effectiveness of SNS use in academia, students' satisfaction, and SNS use impact on students’ performance.

This study's results could be limited due to the quantitative nature of the exploration of students' feelings and worries. Thus, it would be more informative if a qualitative phenomenological study is conducted to explore the unique psychological experiences of students during the COVID-19 pandemic. In addition, the time spent on SNS was not assessed in this study; we recommend that future studies should assess the time spent on SNS usage activities because it is an important factor in the psychological distress experiences of the students. Prior to COVID-19, studies (Turel and Qahri-Saremi, 2016; Satici and Uysal 2015) showed that improper use of the SNS was negatively correlated with the psychological distress symptoms of students. Furthermore, because of our hurriedness toward assessing the psychological status of the students during this pandemic, we omitted other factors that should be explored such as spousal relationships, household arrangements, economic status, and academic performance of the students which may aggravate the psychological distress among undergraduate students. Future studies that examine the association of these factors with the psychological status of undergraduate students during the COVID-19 pandemic are recommended.

## 5 Conclusions

As expected, the largest group of the surveyed students were suffering from depression, anxiety, and stress symptoms. Female students suffered from stress and anxiety more than males. However, the academic usage of the SNS was associated with lessened psychological distress. On the other hand, the use of SNS for entertainment purposes was associated with increased symptoms of stress, and anxiety. This study recommends that educators should consider using SNS to deliver the academic content of their courses. Academic help and consultation are also recommended. In addition, educators are encouraged to direct their students toward the most accurate and up to date COVID-19 resources. Finally, further studies are highly recommended since the story of COVID-19 and its impact on the psychological status of the students is unfolded yet.

## Declarations

### Author contribution statement

T. Al-Dwaikat: Conceived and designed the experiments; Performed the experiments; Analyzed and interpreted the data; Wrote the paper.

M. Aldalaykeh: Performed the experiments; Contributed reagents, materials, analysis tools or data.

W. Ta'an: Analyzed and interpreted the data; Contributed reagents, materials, analysis tools or data; Wrote the paper.

M. Rababa: Analyzed and interpreted the data; Wrote the paper.

### Funding statement

This research did not receive any specific grant from funding agencies in the public, commercial, or not-for-profit sectors.

### Data availability statement

Data will be made available on request.

### Declaration of interests statement

The authors declare no conflict of interest.

### Additional information

No additional information is available for this paper.
